# Do Dietary Supplements and Nutraceuticals Have Effects on Dental Implant Osseointegration? A Scoping Review

**DOI:** 10.3390/nu12010268

**Published:** 2020-01-20

**Authors:** Livia Nastri, Antimo Moretti, Silvia Migliaccio, Marco Paoletta, Marco Annunziata, Sara Liguori, Giuseppe Toro, Massimiliano Bianco, Gennaro Cecoro, Luigi Guida, Giovanni Iolascon

**Affiliations:** 1 Department of Medical and Surgical Specialties and Dentistry, University of Campania “Luigi Vanvitelli”, 80138 Naples, Italy; livia.nastri@unicampania.it (L.N.); paolettamarco@libero.it (M.P.); marco.annunziata@unicampania.it (M.A.); s.liguori@hotmail.it (S.L.); peppetoro@msn.com (G.T.); massimiliano.bianco27@gmail.com (M.B.); gennarocecoro@gmail.com (G.C.); luigi.guida@unicampania.it (L.G.); giovanni.iolascon@gmail.com (G.I.); 2 Department of Movement, Human and Health Sciences, Unit Endocrinology, University Foro Italico, 00135 Rome, Italy; silvia.migliaccio@uniroma4.it

**Keywords:** dietary supplements, dental implants, osseointegration, vitamin D, magnesium, resveratrol, ascorbic acid, zinc, calcium, bone

## Abstract

Several factors affect dental implant osseointegration, including surgical issues, bone quality and quantity, and host-related factors, such as patients’ nutritional status. Many micronutrients might play a key role in dental implant osseointegration by influencing some alveolar bone parameters, such as healing of the alveolus after tooth extraction. This scoping review aims to summarize the role of dietary supplements in optimizing osseointegration after implant insertion surgery. A technical expert panel (TEP) of 11 medical specialists with expertise in oral surgery, bone metabolism, nutrition, and orthopedic surgery performed the review following the PRISMA-ScR (Preferred Reporting Items for Systematic Reviews and Meta-Analyses Extension for Scoping Reviews) model. The TEP identified micronutrients from the “European Union (EU) Register of nutrition and health claims made on foods” that have a relationship with bone and tooth health, and planned a PubMed search, selecting micronutrients previously identified as MeSH (Medical Subject Headings) terms and adding to each of them the words “dental implants” and “osseointegration”. The TEP identified 19 studies concerning vitamin D, magnesium, resveratrol, vitamin C, a mixture of calcium, magnesium, zinc, and vitamin D, and synthetic bone mineral. However, several micronutrients are non-authorized by the “EU Register on nutrition and health claims” for improving bone and/or tooth health. Our scoping review suggests a limited role of nutraceuticals in promoting osseointegration of dental implants, although, in some cases, such as for vitamin D deficiency, there is a clear link among their deficit, reduced osseointegration, and early implant failure, thus requiring an adequate supplementation.

## 1. Introduction

Osseointegration is defined as “a process whereby a clinically asymptomatic rigid fixation of alloplastic materials is achieved and maintained in bone during functional loading” [1]. Osseointegration is involved in dental implants healing, thus leading to a functional unit that may rehabilitate one or more missing teeth, supporting dental prosthesis.

In addition to key factors that affect the osseointegration, such as the surgical technique, bone quality and quantity, postoperative inflammation or infection, smoking habits, and implant material and surface [2,3,4,5,6,7], other factors should be taken into account, including the immunological and nutritional status of the host. Alongside the promotion of a healthy diet, such as the Mediterranean one, to achieve a desirable general health status, recently, increasing attention was paid to promoting the consumption of micronutrients that could have benefits on health and resistance to diseases [8].

Several micronutrients affecting bone metabolism were demonstrated to have an influence on skeletal system; in particular, calcium, fluorides, magnesium, potassium, vitamin B6, vitamin D, and zinc positively influence bone health, reducing the risk of fracture [9]. In addition, fat-, carbohydrate-, and cholesterol-rich diets and reduced calcium intake exhibit detrimental influences on jaw bone and alveolar bone [10]. Therefore, a specific diet regimen and micronutrients might play a key role in the different phases of dental implant osseointegration.

Recent evidence demonstrated that some nutritional regimens directly influence alveolar bone parameters in experimental models of periodontitis [11,12,13], orthodontic tooth movement [14], and bone repair after tooth extraction [15]. In particular, it was demonstrated that diet (in its different meanings of macro- and micronutrients) can affect the healing of the alveolus after tooth extraction, influencing both the morphology and the quality of alveolar bone [15].

Bone tissue repair mechanisms and bone metabolism are strongly influenced by nutritional aspects and are crucial to obtaining proper bone restoration optimizing osseointegration processes.

The aim of this scoping review is to summarize the state of the art regarding the role of micronutrients, currently available in nutraceuticals or dietary supplements, on dental implantology, highlighting which of them, supported by evidence-based medicine (EBM), might effectively have an influence on the achievement and the maintenance of osseointegration after implant insertion surgery.

## 2. Materials and Methods

In performing this scoping review, we followed the PRISMA-ScR (Preferred Reporting Items for Systematic Reviews and Meta-Analyses Extension for Scoping Reviews) model [16].

As a first step, a technical expert panel (TEP) consisting of 11 medical specialists was established. In particular, the TEP was composed of two oral surgeons with expertise in osseointegrated dental implants, two periodontists with expertise in peri-implant oral tissues physiology and pathology, three bone specialists, two experts on scoping review methodology, one nutritionist, and one orthopedic surgeon.

### 2.1. Search Strategy

The TEP selected micronutrients from the “European Union (EU) Register of nutrition and health claims made on foods” that have a relationship with bone and tooth health, included in dietary supplements and nutraceuticals. Therefore, the TEP planned a research on PubMed (Public MedLine, run by the National Center of Biotechnology Information, NCBI, of the National Library of Medicine of Bethesda, Bethesda, MD, USA), selecting micronutrients as MeSH (Medical Subject Headings) terms; to each of them, the following terms were added to run the PubMed Search Builder: “dental implants”, “osseointegration”. For example: (“Vitamin D” [Mesh]) AND “Dental Implants” [Mesh]) (see Supplementary Materials, Table S1).

### 2.2. Study Selection

According to the objective of the study, the TEP defined the characteristics of the sources of evidence, considering for eligibility any researches published in medical literature in the last 10 years (last update on 16 October 2019), including only those in the English language.

### 2.3. Data Extraction and Quality Assessment

All types of studies were included in our scoping review, both pre-clinical (in vitro and animal studies) and clinical studies. Methodological quality assessment was made according to the EBM pyramid: meta-analysis, systematic review, randomized controlled trial (RCT), cohort study, case–control study, case series, and case report.

Finally, the TEP summarized the resulting micronutrients with effective and safe daily doses that improve bone and tooth health.

## 3. Results

From the micronutrients listed in the “EU Register of nutrition and health claims made on foods”, the TEP selected the following 18 nutraceuticals that may have influence on bone and teeth: calcium, fluorides, magnesium, potassium, resveratrol, vitamin C (ascorbic acid), vitamin D, vitamin E (alpha-tocopherol), vitamin K2 (menaquinone-7, MK7), zinc, vitamin A, vitamin B1 (thiamine), vitamin B2 (riboflavin), vitamin B3 (niacinamide), vitamin B5 (pantothenic acid), vitamin B6, vitamin B7 (biotin), and vitamin B12 (Table 1). However, according to the “EU Register of nutrition and health claims made on foods”, fluoride is non-authorized for supporting bone mineralization, and potassium is non-authorized for maintaining tooth mineralization, whereas vitamin B2, vitamin E, vitamin A, vitamin B1, vitamin B2, vitamin B3, vitamin B6, vitamin B7, and vitamin B12 are non-authorized for both functions. Moreover, potassium and zinc are not considered to influence tooth metabolism, while vitamin K2, resveratrol, and vitamin B5 are not recommended for bone and tooth metabolism according to the “EU Register of nutrition and health claims made on foods”. Among these substances, we found studies concerning nutraceuticals and dental implants or osseointegration only for vitamin D, magnesium, resveratrol, vitamin C, a mixture of calcium, magnesium, zinc, and vitamin D, and synthetic bone mineral (a supplement containing calcium, phosphate, magnesium, zinc, fluoride, and carbonate).

**Figure 1:**
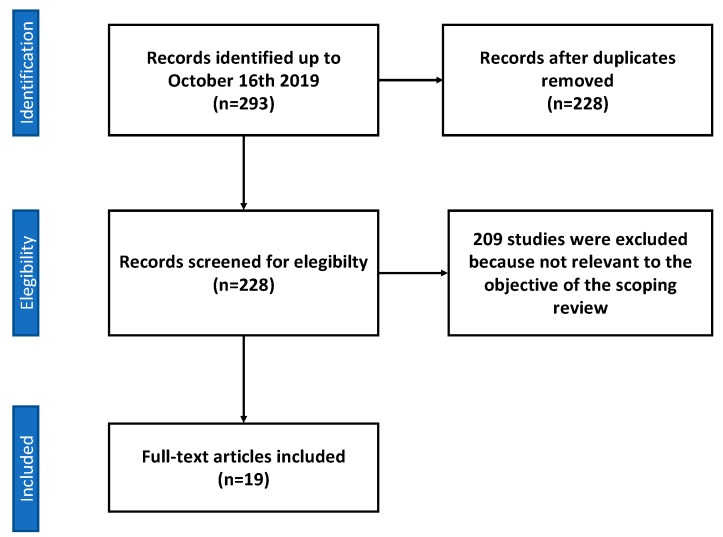
Flow diagram of sources selection process.

In particular, we included 11 studies concerning vitamin D, of which five were clinical studies (three retrospective studies, one case series, and one case report), and six were preclinical studies on animals: two preclinical studies on animals concerning magnesium, two preclinical studies on animals for resveratrol, one preclinical study on animals concerning the supplementation with a combination of calcium, magnesium, zinc and vitamin D, two preclinical studies on animals concerning synthetic bone mineral (composed by dicalcium phosphate dihydrate and magnesium and zinc chlorides), and one clinical study concerning vitamin C supplementation (Figure 1, Table 2).

### 3.1. Vitamin D

#### 3.1.1. Animal Studies

In our scoping review, we included six preclinical studies on animal models, more precisely, on rats.

Liu et al., in 2014 [34], found that vitamin D supplementation in rats affected by chronic kidney disease (CKD) improved bone-to-implant contact (BIC) compared to CKD rats that did not receive vitamin D, making this finding comparable to that of rats without CKD. Also, the bone volume in the circumferential zone within 100 mm of the implant surface increased after vitamin D administration. At two weeks, the push-in test showed significantly better results for the vitamin D-treated group compared to untreated CKD mice.

Zhou et al., in 2012 [35], demonstrated that vitamin D supplementation in osteoporotic rats, eight weeks after implantation, improved bone volume, osseointegration, mean trabecular number, mean trabecular thickness, and trabecular connective density, while it decreased trabecular separation, as well as increased bone area density, BIC, and the maximal push-out force.

Wu et al., in 2012 [36], inserted titanium implants in diabetic rats and evaluated the effects of different kinds of diabetes therapies. The combined therapy with insulin and vitamin D showed the best effects on osseointegration, bone volume, mean trabecular thickness, mean trabecular number, connective density, mean trabecular separation, push out force, shear strength, BIC, and bone area ratio. Treatments with vitamin D or insulin only showed better results compared to untreated diabetic rats, but worse than the combined therapy. All the parameters listed above, in the combined treatment group, resulted similar to those of the control healthy group.

Akhavan et al., in 2012 [37], evaluated the effects of vitamin D supplementation on BIC in diabetic rats compared to a placebo group. At three weeks, the vitamin D group showed higher values of BIC compared to the placebo group, and also at six weeks, even if in a non-statistically significant way, leading the authors to conclude that vitamin D seems to not have an effect on the osseointegration of implants in diabetic rats.

Dvorak et al., in 2012 [38], showed that, in osteoporotic rats, a vitamin D depletion led to a significant decrease in BIC in the cortical area. In rats that received a vitamin D-free diet, followed by vitamin D repletion, no significant difference could be found compared to the control group that received a standard vitamin D diet.

Kelly et al., in 2008 [39], found that vitamin D deficiency, 14 days after implantation, led to a lower push-in test and a decreased BIC compared to a normal vitamin D status.

#### 3.1.2. Clinical Studies

The clinical studies on vitamin D that we included in this scoping review were three retrospective studies, one case series, and one case report.

From the retrospective studies of Mangano et al. of 2016 [31] and 2018 [29], it emerged that, in patients with vitamin D deficiency, there were a higher percentage of early dental implant failures (failures that occurred before prosthesis positioning, EDIF). However, although there was a clear trend toward an increased incidence of EDIF with lower serum 25(OH)D, no statistically significant difference was found among the three groups with different vitamin D status.

In the retrospective study of Wagner et al. of 2017 [30], osteoporosis was shown to have a significant negative influence on marginal bone loss around implants, but vitamin D significantly affected the marginal bone loss at the mesial and distal implant aspect, showing beneficial effects on the peri-implant bone formation.

Fretwurst et al., in 2016 [32], reported two cases of implant failures occurring within 15 days of surgery in patients with vitamin D deficiency; in one patient, there were even two consecutive implant failures. In both patients, after vitamin D supplementation, implants were placed successfully. The authors also noticed that failures were sometimes associated with pain and discomfort in vitamin D-deficient patients.

Also, Bryce and Macbeth, in 2014 [33], reported a case of missed osseointegration in a patient affected by severe vitamin D deficiency.

#### 3.1.3. Magnesium

We included two animal studies that evaluated the effects of magnesium deficiency on osseointegration of titanium implants. The deficiency of magnesium led to lower cortical bone thickness, lower values of removal torque of the implants, and lower bone mineral density (BMD) [40,41]. In detail, Bellucci et al., in 2011 [40], found that a 90% reduction of magnesium intake, 90 days after implant insertion, led to lower BMD values. In the magnesium reduction group, upper and lower cortical thicknesses were significantly reduced, as well as the removal torque of the implants. On the other hand, the radiographic bone density and cortical thickness around the implants resulted similar between the two groups.

Del Barrio et al., in 2010 [41], reported that only a 90% reduced magnesium intake resulted in low BMD after implant insertion compared to both a 75% magnesium intake reduction and a normal magnesium intake.

#### 3.1.4. Resveratrol

We found two animal studies that evaluated the effects of resveratrol intake on the osseointegration of titanium implants.

In 2018, Ribeiro et al. [42] demonstrated that supplementation of resveratrol led to an improvement in counter-torque and BIC in rats exposed to cigarette smoking, compared to rats exposed to cigarette smoking but receiving placebo. This finding seems quite relevant, considering that detrimental effects of smoking on oral health in terms of increased postoperative infections and marginal bone loss in patients receiving dental implants are well established [48,49,50]. Also, Casarin et al., in 2014 [43], demonstrated that resveratrol intake had positive effects on the biomechanical retention of the implants, because there were significantly higher average counter-torque values for implant removal in rats that received resveratrol.

#### 3.1.5. Mixtures of Micronutrients

Pimentel et al., in 2016 [44], evaluated the effects of a mixture of calcium, magnesium, zinc, and vitamin D on rats that received titanium implants. They found that there was no statistically significant difference among the counter-torque values for implant removal, bone volume, and BIC in the placebo group when compared to the micronutrient group.

Takahashi et al., in 2016 [45], evaluated the effects of supplementation with synthetic bone mineral (SBM), a mixture of calcium phosphate dihydrate and magnesium and zinc chlorides, on titanium implants in osteoporotic rats. They found significantly higher bone volume and lower bone surface ratio in the SBM group. Moreover, the trabecular thickness increased significantly from two to four weeks after implant insertion in treated group, while the improvement of the same parameters was not significant in the control group. Also, other histomorphometric parameters significantly improved in SBM group, such as the trabecular star volume, although the between-group difference in terms of trabecular number was not significant. Finally, rats receiving SBM showed enhanced bone formation, evaluated by micro-computed tomography (micro-CT), both at two and at four weeks compared to rats fed without SBM.

Also, Watanabe et al., in 2015 [46], evaluated the effects of SBM on osseointegration in rats. They found that pull-out strength in the treated group was six times higher than in the control group two weeks after implantation and twice higher at four weeks. The BMD in the SBM group was approximately double compared to the control group at two weeks and more than double at four weeks. BMD color imaging showed that the control group colors mainly ranged from blue to yellow at two and four weeks after implantation, while the SBM group mainly occupied the orange and red end of the spectrum at two and four weeks after implantation. Given that blue and light blue, green and yellow, and orange and red represent low, medium, and high BMD, respectively, the BMD color imaging indicated that peri-implant bone had a higher BMD in the SBM group than in the control group. Fluorescence microscopy imaging of the control group revealed no green fluorescence at two weeks after implantation. However, green fluorescence was clearly observed in the SBM group at two and four weeks after implantation, while irregular bands appeared around the implants in the control group at four weeks.

### 3.2. Vitamin C

Li et al., in 2018 [47], evaluated the effects of vitamin C supplementation on four populations: patients receiving dental implants by guided bone regeneration (GBR), patients treated with Bio-Oss collagen, patients with chronic periodontitis receiving dental implants, and a control group without any bone grafting or periodontal disease. The authors found that vitamin C supplementation improved postoperative wound healing following dental implant surgery in patients with chronic periodontitis and in those treated with GBR or Bio-Oss collagen grafts. However, vitamin C supplementation was ineffective in decreasing the postoperative pain associated with dental implant surgery.

## 4. Discussion

To the best of our knowledge, this is the first scoping review to investigate the putative role of dietary supplements in affecting bone structural and mechanical properties involved in dental implant osseointegration, as well as in improving clinical outcomes, such as the maintenance of peri-implant tissue health and implant success rate.

The Federal Food, Drug, and Cosmetic Act defines a dietary supplement as a product that is intended to supplement the diet, which bears or contains one or more ingredients including a vitamin, mineral, herb, and amino acid, or a concentrate, metabolite, constituent, extract, or combinations of these [50]. The term “nutraceutical” was coined by Stephen De Felice to define “food (or parts of a food) that provides medical or health benefits, including the prevention and/or treatment of a disease”, by the fusion of the words “nutrition” and “pharmaceutical”, commonly used in marketing with no regulatory legal definition [51]. Ten years later, nutraceuticals are defined as dietary supplements that include a concentrated form of a presumed bioactive substance, originally derived from a food, but present in a non-food matrix, and used to maintain or improve health status in dosages exceeding those obtainable from conventional foods [52].

It should be stressed, however, that there is no consensus with regard to “nutraceutical” definition or similar terms. Aronson recently considered that the term “nutraceuticals” is too vague and should be abandoned, even if he did not propose any robust alternatives [53].

According to the recent data of the United States (US) Centers for Disease Control and Prevention’s National Health and Nutrition Examination Survey (NHANES), more than 25% of the US population had an insufficient intake of vitamins A, C, D, and E, as well as calcium, magnesium, and potassium in their diet; thus, the modern diet of Western countries does not seem to have an adequate intake of micronutrients. It was reported that micronutrient deficiencies affect around two billion people worldwide [54]. However, a consensus about the use of these substances, particularly concerning the adequate amount and safety, is not yet reached, even if they are supposed to have multiple physiological beneficial effects.

Several micronutrients are hypothesized to have an influence on skeletal system, particularly on jaw bone and alveolar bone [9] and on dental implant osseointegration. However, according to our findings, very few elements (i.e., vitamin D, magnesium, resveratrol, and vitamin C) were the matter of previous investigations on their role in dental implant osseointegration. Available data suggest that severe vitamin D deficiency or even the presence of established osteoporosis led to a higher implant failure rate [29,31] or to a worse BIC [34,35]. In osteoporotic rats, vitamin D depletion led to a significant decrease in BIC in the cortical area. Moreover, rats that received vitamin D showed a similar BIC to the control group [34]. Animal studies on vitamin D and osseointegration confirmed that the early stages of bone healing could be significantly influenced by vitamin D status [35,36,37,38,39,40]. In humans, Mangano et al. [29,31] reported a clear trend toward an increased incidence of early implant failures within the group with lower serum 25(OH)D levels. In particular, the authors reported 11.1% EDIF in patients with serum 25(OH)D < 10 ng/mL (severe vitamin D deficiency), 4.4% for those with 25(OH)D between 10 and 30 ng/mL, and 2.9% in patients with normal vitamin D status.

Moreover, Wagner et al. [30] showed that osteoporosis has a significant negative influence on marginal bone loss around implants and that vitamin D supplementation counteracts the marginal bone loss, with overall results of beneficial effects on the peri-implant bone formation.

Vitamin D deficiency commonly occurs in the general population. This hormone has a crucial function in skeletal mineralization, but also plays an important role in immunity and inflammatory response, increasing anti-inflammatory cytokines and decreasing pro-inflammatory ones [55].

Bashutski et al. [56] showed that, in vitamin D-deficient individuals, minimal benefits could be obtained from periodontal surgery along with an impaired post-surgical healing. Vitamin D could have other effects on osseointegration that are more related to soft tissue healing and marginal seals around implants, together with an effect on resistance against bacterial infections of the peri-implant sulcus. Also, topical applications of vitamin D were used for implant coating, showing some beneficial effects in animals, such as a reduction in crestal bone loss and an increase of BIC [57]. However, several critical issues persist regarding the use of vitamin D in enhancing osseointegration, particularly concerning its mechanism(s) of action, the influence of different serum 25(OH)D levels, and the recommended dosages required to significantly improve dental implant success rate.

Also, vitamin C deficiency may have a role in tissue healing and stability around dental implants. This micronutrient plays an important role in the biosynthesis of collagen, which is an important component of connective tissue of the gingiva, peri-implant mucosa, and alveolar bone [58]. These effects were confirmed by Li et al. [47], who found that vitamin C supplementation improved postoperative wound healing following dental implant surgery. Moreover, protective effects of this intervention on bone health could be expected, as vitamin C could hinder the effects of oxidative stress in promoting bone resorption and consequently reducing bone strength [59], although this hypothesis is not yet confirmed. However, the role of vitamin C supplementation in the general population, as well as in patients receiving dental implants, might be significantly affected by lifestyle, including smoking habits and diet, two factors that affect wound healing times. Furthermore, plasma ascorbic acid concentrations are not reported in clinical practice [60].

With regard to resveratrol, Casarin et al. [43] investigated its role on bone healing of calvarial defects in rats through messenger RNA (mRNA) quantification of bone morphogenetic protein (BMP)-2, BMP-7, osteopontin (OPN), bone sialoprotein (BSP), osteoprotegerin (OPG), and receptor activator of nuclear factor kappa B (NF-κB) ligand (RANKL). Gene expression analysis showed a higher expression of *BMP-2* (*p* = 0.011), *BMP-7* (*p* = 0.049), and *OPN* (*p* = 0.002) genes in the resveratrol-fed group than in the control group.

Ribeiro et al. [42] reported encouraging data about biomechanical retention and peri-implant bone formation in resveratrol-fed rats exposed to cigarette smoking inhalation, supporting a positive role of this substance in controlling different osteogenic mechanisms. Their gene expression analysis demonstrated that lower RANKL/OPG levels were detected in rats receiving resveratrol, as well as in non-smoking animals, when compared to animals exposed to smoking and receiving placebo. Both studies on resveratrol confirmed the substantial improvement in implant stability, by modulating the expression of genes involved in bone regulatory processes. However, the main limitation of findings supporting resveratrol, as well as magnesium, is the availability of animal studies only.

However, considering the results of our research, several micronutrients are non-authorized or even not considered by the “EU Register on nutrition and health claims” on the basis of current scientific evidence.

Major nutrients involved in skeletal health include calcium, phosphorus, vitamin D, magnesium, and potassium, but other micronutrients and trace elements such as boron, selenium, iron, zinc, and copper also impact bone metabolism. Information on the influence of such “minor” elements coming from studies on nutrient depletion and studies on osseointegration is still lacking.

## 5. Conclusions

Our scoping review overall demonstrated a lack of data about the effects of micronutrients and nutraceuticals on osseointegration of dental implants, although, for some of them, such as vitamin D, there was a clear association among their deficit, reduced osseointegration, and increased early implant failure incidence in both animal and human studies.

Some micronutrient deficiencies are supposed to increase oxidative stress and inflammation and to affect collagen structure and bone mineralization. For these reasons, it would be desirable that further studies investigate the hypothesis of an influence of micronutrients and nutraceuticals on dental implant osseointegration and long-term success, as well as the opportunity of a diet integration to enhance peri-implant wound healing, bone healing, and peri-implant tissue stability. However, data for many micronutrients that might modulate bone metabolism are lacking, and dosing regimens for dietary supplements that improve dental implant osseointegration are not defined according to available findings; furthermore, safety issues remain to be carefully investigated. In conclusion, our findings support an ancillary role of vitamin D, in patients with vitamin D deficiency, as well as vitamin C supplementation, in facilitating the success of the dental implant surgery.

## Figures and Tables

**Table 1:** Effects of selected micronutrients on bone and tooth health.

Nutrient or Non-Nutrient Compound	Effect
Calcium	99% of calcium in the body is in the form of hydroxyapatite, which is bone and tooth mineral [17]. Relationship between calcium and maintenance of normal bone and tooth assessed with a favorable outcome (European Food Safety Authority (EFSA) opinion).
Fluorides	Stimulates osteoblast growth and bone formation, increasing bone mineral density (BMD) [18], and supports tooth mineralization (EFSA opinion).
Magnesium	Essential for the conversion of vitamin D into its active form and necessary for calcium absorption and metabolism [19], and maintenance of normal bone and teeth (EFSA opinion).
Potassium	Potassium citrate helps maintain acid–base balance and support bone health, counteracting bone resorption [20]. However, a cause-and-effect relationship is not established between the dietary intake of potassium salts of citric acid and maintenance of normal bone (EFSA opinion).
Resveratrol	Active substance found in food, such as red grapes, peanuts, and berries, with anti-inflammatory and antioxidant effects; it additionally provides an inhibitory effect on osteoclast differentiation and potentially induces bone formation [21] (EFSA opinion about bone and tooth health not available).
Vitamin C (ascorbic acid)	Enhances osteoblastogenesis and inhibits osteoclastogenesis via Wnt/β-catenin signaling [22]. Vitamin C contributes to normal function of bones and teeth (EFSA opinion).
Vitamin D	Modulates calcium and phosphate metabolism; it promotes growth, bone mineralization of the skeleton and teeth [17], and maintenance of normal bone and teeth (EFSA opinion).
Vitamin E (alpha-tocopherol)	Reduces the expression of receptor activator of nuclear factor kappa B (NF-κB) ligand (RANKL) in osteoblasts and inhibits osteoclastogenesis [23]. However, a cause-and-effect relationship is not established between the dietary intake of vitamin E and maintenance of normal bone and teeth (EFSA opinion).
Vitamin K2 (MK7)	Stimulates osteoblasts differentiation, protects these cells from apoptosis [24], and maintains normal bone (EFSA opinion).
Zinc	Stimulates osteoblast proliferation, differentiation, and mineralization, which may facilitate bone formation [25,26] and maintain normal bone (EFSA opinion).
Vitamin A	Increases the effect of bone morphogenetic proteins (BMPs) on osteogenic differentiation [27]. A cause-and-effect relationship is not established between the dietary intake of vitamin A and maintenance of normal bone and teeth (EFSA opinion).
B Vitamins	Deficiency in folic acid and vitamins B6 and B12 can result in increased serum homocysteine that leads to endothelial dysfunction (decreased bone blood flow) and enhanced osteoclast activity (bone resorption). Moreover, hyperhomocysteinemia interferes with cross-linking of collagen (altered bone matrix) [28]. However, a cause-and-effect relationship is not established between the dietary intake of B vitamins and maintenance of normal bone and teeth (EFSA opinion).

**Table 2:** Relevant data from each study included in the scoping review.

Author, Year	Nutraceutical Compound	Study Design/Experimental Model	Main Aim	Results
Mangano et al., 2018 [29]	Vitamin D	Retrospective study	To investigate the correlation between serum levels of vitamin D and early dental implant failure	In patients with serum levels of vitamin D < 10 ng/mL, there were 11.1% early dental implant failures (EDIF; failures that occurred before prosthesis positioning), 4.4% EDIFs in patients with vitamin D levels between 10 and 30 ng/mL, and 2.9% EDIFs in patients with levels > 30 ng/mL. No statistically significant correlation was found between EDIF and vitamin D serum levels, but a clear trend toward an increased incidence of EDIF with lowering of serum vitamin D levels was reported.
Wagner et al., 2017 [30]	Vitamin D	Retrospective parallel group	To evaluate the influence of osteoporosis on the marginal peri-implant bone level	Osteoporosis was shown to have a significant negative influence on the marginal bone loss (MBL) at the mesial and the distal implant aspect. Vitamin D positively and significantly affected the MBL, showing beneficial effects on the peri-implant bone formation.
Mangano et al., 2016 [31]	Vitamin D	Retrospective study	To investigate the correlation between early dental implant failure and low serum levels of vitamin D	There were 9% EDIFs in patients with serum levels of vitamin D < 10 ng/mL, 3.9% EDIFs in patients with vitamin D levels between 10 and 30 ng/mL, and 2.2% EDIFs in patients with vitamin D levels > 30 ng/mL. Although there was an increasing trend in the incidence of early implant failures with the worsening of vitamin D deficiency, the difference between these 3 groups was not statistically significant.
Fretwurst et al., 2016 [32]	Vitamin D	Case series	To evaluate the correlation between vitamin D deficiency and early implant failure	After vitamin D supplementation, implant placement was successful in 2 patients with previous early implant failures.
Bryce & Macbeth, 2014 [33]	Vitamin D	Case report	To investigate the influence of vitamin D deficiency in the osseointegration process of a dental implant	Authors reported a case of a patient that received dental extraction and the insertion of an immediate implant that failed to osseointegrate. Medical investigations revealed that he was severely vitamin D-deficient and that this may have contributed to the implant failure.
Liu et al., 2014 [34]	Vitamin D	Animal study	To investigate the effect of Vitamin D supplementation on implant osseointegration in CKD mice.	In rats with chronic kidney disease (CKD), vitamin D supplementation led to bone-to-implant contact rate (BIC) and bone volume/total volume levels higher than the CKD group without supplementation and comparable to rats without CKD. Also, at the push-in test, the CKD + vitamin D group had better results than the CKD group, which were comparable to the control group.
Zhou et al., 2012 [35]	Vitamin D	Animal study	Investigate the effects of 1,25(OH)2D3 on implant osseointegration in osteoporotic rats	Vitamin D supplementation in osteoporotic rats led to formation of more cancellous bone around implants, an increase of bone volume by 96.0% in terms of osseointegration, by 94.4% in terms of mean trabecular number, by 112.5% in terms of mean trabecular thickness, by 51.8% in terms of trabecular connective density, and by 38.0% in terms of connective density, as well as a decrease in terms of trabecular separation by 39.3%. Vitamin D increased bone area density by 1.2-fold and bone-to-implant contact by 1.5-fold and increased the maximal push-out force by 2.0-fold.
Wu et al., 2012 [36]	Vitamin D	Animal study	Effect of insulin and vitamin D3 on implant osseointegration in diabetic mellitus rats	Vitamin D and insulin combined treatment of diabetic rats led to an improvement of bone volume per total volume, percentage of osseointegration, mean trabecular thickness, mean trabecular number, connective density, maximal push-out force, and ultimate shear strength, BIC, and bone area ratio (BA), while the mean trabecular separation decreased. These indexes showed values comparable to those of healthy control rats.
Akhavan et al., 2012 [37]	Vitamin D	Animal study	Compare the effect of vitamin D administration on bone to implant contact in diabetic rats	At the histological analysis 3 weeks after implant insertion, diabetic rats reported a BIC level of 44 ± 19, while diabetic rats receiving vitamin D had a level of 57 ± 20. At 6 weeks, the control group reported BIC level of 70 ± 29 and the vitamin D group had a level of 65 ± 22. Considering these results, vitamin D seems not to have an effect on osseointegration of implants in diabetic rats.
Dvorak et al., 2012 [38]	Vitamin D (deficiency)	Animal study	Impact of vitamin D supplementation on the process of osseointegration	Vitamin D depletion in ovariectomized rats led to a significant decrease in bone-to-implant contact in the cortical area compared to rats fed with a standard vitamin D diet, while no significant reduction in BIC was observed in the medullar and the periosteal compartment.
Kelly et al., 2008 [39]	Vitamin D (deficiency)	Animal study	To evaluate the effect of a common deficiency of vitamin D on implant osseointegration in the rat model	Vitamin D deficiency in rats, 14 days after implant insertion, led to a lower push-in test and a lower BIC, compared to rats without deficiency. SEM analyses showed that the calcified tissues after push-in test, in the vitamin D deficiency groups, fractured between the implant and the surrounding tissue, resulting in exposed implant surface.
Belluci et al., 2011 [40]	Magnesium	Animal study	To evaluate the effect of magnesium dietary deficiency on bone metabolism and bone tissue around implants with established osseointegration	Rats fed with a diet with 90% magnesium reduction presented loss of systemic bone mass, decreased cortical bone thickness, and lower values of removal torque of the implants.
Del Barrio et al., 2010 [41]	Magnesium	Animal study	To evaluate the effect of severe magnesium dietary deficiency on systemic bone density and biomechanical resistance of bone tissue to the removal torque of osseointegrated implants	Magnesium intake reduction of 90% in diet of rats led to a statistically lower removal torque of the implants compared to rats fed with the recommended magnesium content, while no difference was demonstrated between the group with a 75% magnesium reduction and the control group.
Ribeiro et al., 2018 [42]	Resveratrol	Animal study	To investigate the effect of resveratrol on peri-implant repair, and its influence on bone-related markers in rats	Systemic assumption of resveratrol positively affected biomechanical retention of titanium implants, measured as torque removal values, and determined a higher BIC in smoking rats, when compared to smoking + placebo rat group.
Casarin et al., 2014 [43]	Resveratrol	Animal study	To investigate the effect of resveratrol on bone healing and its influence on the gene expression of osteogenic markers	Resveratrol increased the counter-torque values of implant removal when compared to placebo therapy and increased bone healing of critical size defects in rats.
Pimentel et al., 2016 [44]	Calcium, magnesium, zinc, and vitamin D3	Animal study	To investigate the effect of micronutrients supplementation on the bone repair around implants	Rats receiving calcium, magnesium, zinc, and vitamin D intake for 30 days after implant insertion showed counter-torque values with no statistical difference compared to rats that received a placebo solution. Neither bone volume per total volume nor BIC showed a statistically significant difference between the 2 groups.
Takahashi et al., 2016 [45]	Synthetic bone mineral (dicalcium phosphate dihydrate + magnesium and zinc chlorides)	Animal study	To investigate whether oral intake of synthetic bone mineral improves peri-implant bone formation and bone micro architecture	Synthetic bone mineral (SBM; a mixture of dicalcium phosphate dihydrate and magnesium and zinc chlorides) intake led to a significantly higher bone volume per total volume, trabecular thickness, trabecular star volume compared to rats fed without SBM. The bone surface ratio of the rats that were fed with SBM was significantly lower than that of the rats fed without SBM. The trabecular number of the rats fed with SBM was not significantly increased compared to rats fed without SBM. Rats fed without SBM had no bone formation at 2 weeks, while bone formation was clearly observed in rats fed with SBM at 2 and 4 weeks after implantation. In rats fed without SBM at 4 weeks after implantation, irregular bone bands around the implants were observed.
Watanabe et al., 2015 [46]	Synthetic bone mineral (dicalcium phosphate dihydrate + magnesium and zinc chlorides)	Animal study	To investigate the effect of synthetic bone mineral in accelerating peri-implant bone formation	Pull-out strength was greatly higher in the SBM group compared to control group at 2 and 4 weeks. Bone mineral density was approximately double in the SBM group compared to control group both at 2 and 4 weeks, and this result was confirmed also by bone mineral density (BMD) color imaging. Microscopy observation showed green fluorescence in the SBM group at 2 and 4 weeks and only at 4 weeks in the control group.
Li et al., 2018 [47]	Vitamin C	Parallel group	To explore the effects of vitamin C supplementation in wound healing, following the placement of dental implants with or without bone grafts and patients with chronic periodontitis	Patients that received implants with guided bone regeneration (GBR) or with Bio-Oss collagen grafts and received vitamin C supplements, 14 days post-surgery, showed significantly improved wound healing compared with patients receiving the same surgical therapy but without vitamin C supplements. Patients suffering from chronic periodontitis that received implants showed significantly better wound healing at 7 and 14 days when they had vitamin C supplements compared to patients without supplements. Vitamin C showed no postoperative pain relief proprieties in any group.

## Supplementary Materials

The following are available online at https://www.mdpi.com/2072-6643/12/1/268/s1, Table S1: Search strategy.
