# Supplementary material for: Do Dietary Supplements and Nutraceuticals Have Effects on Dental Implant Osseointegration? A Scoping Review
Source: Nutrients. 2020 Jan 20;12(1):268. doi: 10.3390/nu12010268 (PMC7019951; doi:10.3390/nu12010268)
Supplement: Supplementary file 1 [file nutrients-12-00268-s001.pdf]

**Search strategy**
("Vitamin D"[Mesh]) AND "Dental Implants"[Mesh]
("Calcium"[Mesh]) AND "Dental Implants"[Mesh]
("Fluorides"[Mesh]) AND "Dental Implants"[Mesh]
("Magnesium"[Mesh]) AND "Dental Implants"[Mesh]
("Potassium"[Mesh]) AND "Dental Implants"[Mesh]
("Resveratrol"[Mesh]) AND "Dental Implants"[Mesh]
("Vitamin C"[Mesh]) AND "Dental Implants"[Mesh]
("Vitamin E"[Mesh]) AND "Dental Implants"[Mesh]
("Vitamin K2"[Mesh]) AND "Dental Implants"[Mesh]
("Zinc"[Mesh]) AND "Dental Implants"[Mesh]
("Vitamin A"[Mesh]) AND "Dental Implants"[Mesh]
("Vitamin B1"[Mesh]) AND "Dental Implants"[Mesh]
("Vitamin B2"[Mesh]) AND "Dental Implants"[Mesh]
("Vitamin B3"[Mesh]) AND "Dental Implants"[Mesh]
("Vitamin B5"[Mesh]) AND "Dental Implants"[Mesh]
("Vitamin B6"[Mesh]) AND "Dental Implants"[Mesh]
("Vitamin B7"[Mesh]) AND "Dental Implants"[Mesh]
("Vitamin B12"[Mesh]) AND "Dental Implants"[Mesh]
("Vitamin D"[Mesh]) AND "osseointegration"[Mesh]
("Calcium"[Mesh]) AND "osseointegration"[Mesh]
("Fluorides"[Mesh]) AND "osseointegration"[Mesh]
(" Magnesium"[Mesh]) AND "osseointegration"[Mesh]
("Potassium"[Mesh]) AND "osseointegration"[Mesh]
("Resveratrol"[Mesh]) AND "osseointegration"[Mesh]
("Vitamin C"[Mesh]) AND "osseointegration"[Mesh]
("Vitamin E"[Mesh]) AND "osseointegration"[Mesh]
("Vitamin K2"[Mesh]) AND "osseointegration"[Mesh]
("Zinc"[Mesh]) AND "osseointegration"[Mesh]
("Vitamin A"[Mesh]) AND "osseointegration"[Mesh]
("Vitamin B1"[Mesh]) AND "osseointegration"[Mesh]
("Vitamin B2"[Mesh]) AND "osseointegration"[Mesh]
("Vitamin B3"[Mesh]) AND "osseointegration"[Mesh]
("Vitamin B5"[Mesh]) AND "osseointegration"[Mesh]
("Vitamin B6"[Mesh]) AND "osseointegration"[Mesh]
("Vitamin B7"[Mesh]) AND "osseointegration"[Mesh]
("Vitamin B12"[Mesh]) AND "osseointegration"[Mesh]
